# Effect of oxidative stress and calcium deregulation on FAM26F (CALHM6) expression during hepatitis B virus infection

**DOI:** 10.1186/s12879-021-05888-0

**Published:** 2021-02-27

**Authors:** Kehkshan Jabeen, Uzma Malik, Sajid Mansoor, Shaheen Shahzad, Saadia Zahid, Aneela Javed

**Affiliations:** 1 Genomics Research Lab, Department of Biological Sciences, International Islamic University Islamabad, Islamabad, 44000 Pakistan; 2 Department of Healthcare Biotechnology, Atta-ur-Rahman School of Applied Biosciences (ASAB), National University of Sciences and Technology (NUST), H-12 Campus, Islamabad, 44000 Pakistan; 3 Department of Microbiology, Faculty of Life Sciences, University of Central Punjab, Lahore, 54000 Pakistan

**Keywords:** Family with sequence similarity 26 member F (FAM26F), Hepatitis B virus (HBV), Reactive oxygen species (ROS), Calcium deregulation, Calcium chelators, NAC, Predictive marker

## Abstract

**Background:**

Family with sequence similarity 26, member F (FAM26F) is an important innate immunity modulator playing a significant role in diverse immune responses, however, the association of FAM26F expression with HBV infection is not yet known. Thus, the current study aims to explore the differential expression of FAM26F in vitro in HepAD38 and HepG2 cell lines upon HBV infection, and in vivo in HBV infected individuals. The effects of antioxidant and calcium inhibitors on the regulation of FAM26F expression were also evaluated. The expression of FAM26F was simultaneously determined with well-established HBV infection markers: IRF3, and IFN-β.

**Methods:**

The expression of FAM26F and marker genes was analyzed through Real-time qPCR and western blot.

**Results:**

Our results indicate that the differential expression of FAM26F followed the same trend as that of IRF3 and IFN-β. The in vitro study revealed that, in both HBV infected cell lines, FAM26F expression was significantly down-regulated as compared to uninfected control cells. Treatment of cells with N-acetyl-L-cysteine (NAC), EGTA-AM, BAPTA-AM, and Ru360 significantly upregulated the expression of FAM26F in both the cell lines. Moreover, in in vivo study, FAM26F expression was significantly downregulated in all HBV infected groups as compared to controls (*p* = 0.0007). The expression was higher in the HBV recovered cases, probably due to the decrease in infection and increase in the immunity of these individuals.

**Conclusion:**

Our study is the first to show the association of FAM26F with HBV infection. It is proposed that FAM26F expression could be an early predictive marker for HBV infection, and thus is worthy of further investigation.

**Supplementary Information:**

The online version contains supplementary material available at 10.1186/s12879-021-05888-0.

## Background

Hepatitis B virus (HBV) infection is a major health burden worldwide. Globally, there are approximately 257 million people living with HBV infection [1]. HBV infection is characterized by alteration of mitochondrial dynamics, which causes damage to the organelle by depolarizing its membrane, producing reactive oxygen species (ROS), and disturbing calcium homeostasis [2, 3]. Mitochondrial damage and oxidative stress are the main events leading to chronic liver disease [4–6], progressing from hepatitis, to fibrosis, cirrhosis, and finally to hepatocellular carcinoma (HCC) [7]. Globally, HCC is the sixth prevalent cancer and considered the second leading cause of cancer deaths [8, 9].

Family with sequence similarity 26, member F (FAM26F) was initially identified about a decade ago. Since then it has been reported to be a significant player in various regulative functions of the immune system. Various whole transcriptome analyses have demonstrated *FAM26F* to be differentially expressed in several viral (Simian Immunodeficiency Virus and Hepatitis C Virus (HCV)) [10, 11], bacterial *(Septicemic melioidosis, Staphylococcal Superantigens, and Staphylococcal Enterotoxin B)*, [12–14], and parasitic infections like Gastrointestinal Nematode infection [15], in liver transplantation, and in several cancers (breast, mammary gland, cervix, and uterus) [16]. With respect to viral infections, FAM26F has only been studied in SIV [17] and HCV [11]. In SIV-infected Rhesus macaques, pre-infection levels of FAM26F were found to correlate with the overall viral load during the acute phase of infection, recognizing FAM26F as one of the earliest prognostic markers which can give information related to the pace and strength of antiviral immune response [17]. In HCV patients, FAM26F was identified as one out of 91 differentially expressed genes associated with HCV clearance [11].

Due to the versatile nature of a Ca^2+^ signal, one of the mechanisms for viruses to create a permissive cellular environment is the modulation of intracellular Ca^2+^ signaling, which is in turn associated with ROS regulation. HBV expression has been shown to be linked with numerous physiological variations, including up-regulation of ROS levels, and disturbance in calcium homeostasis [18]. Interestingly, FAM26F possesses a conserved Calcium homeostasis modulator (Ca_hom-mod) domain, hence also named as Calcium homeostasis modulator protein 6 (CALHM6) [19]. Recently, FAM26F was found to be functionally related to calcium-binding proteins, and it was proposed that the FAM26F expression is regulated by cytosolic calcium disturbances [20]. Moreover, FAM26F was identified as one of the top classifiers among 371 differentially expressed genes that were functionally linked with oxidative stress and inflammation [21]. FAM26F was also significantly up-regulated along with several chemokines, MHC class I, and MHC class II molecules, in the placental transcriptome of Villitis of unknown etiology (VUE), where its expression increased as a function of the severity of the inflammatory process [22]. All these studies suggest that targeting the ROS and Ca^2+^ pathways can greatly assist us in understanding the unknown association between FAM26F and HBV.

Several inhibitors and chelators of ROS and calcium respectively have been reported in various studies. For example, it has been reported that antioxidant N-acetyl-L-cysteine (NAC) counteracts the oxygen free radical effects [23, 24]. Likewise, calcium signals can be blocked by using calcium chelators and calcium inhibitors. Intracellular [Ca^2+^] chelation is obtained by using BAPTA-AM (1,2-Bis (2-aminophenoxy) ethane-*N,N,N′,N′*-tetraacetic acid tetrakisacetoxymethyl ester) or EGTA-AM (Ethylene-bis (oxyethylenenitrilo) tetraacetic acid Glycol ether diamine tetraacetic acid-acetoxymethyl ester), whereas mitochondrial calcium uptake can be inhibited by using oxygen bridged dinuclear ruthenium amine complex (Ru360). A reversal in ROS production and Ca^2+^ deregulation by using antioxidant and calcium modulators can have a positive effect on HBV clearance or containment, most likely by modulating the expression of FAM26F.

Here, we propose the FAM26F expression to be associated with HBV as it can be hypothesized that the inflammation induced by HBV has some implications on the production of FAM26F*.* Moreover, FAM26F is also considered as an important regulator and activator of the innate immune response involved in important immune signaling cascades. Thus, the current research investigated the changes that occurred in the FAM26F expression in vitro and in vivo upon HBV infection. Additionally, the study probed the effects of ROS and calcium inhibitors on the regulation of FAM26F expression. As the role of IRF3 and IFN-β is significant in mediating antiviral response, and they have also been found to regulate and enhance the FAM26F expression [25, 26]; therefore, IRF3 and IFN-β were also investigated simultaneously with FAM26F, as standards, for the regulation of their expression. The current study will thus be a step forward in highlighting the unseen association of FAM26F with HBV.

## Methods

### Cells lines and plasmids

The HepG2 human hepatoma cells were procured from the American Type Culture Collection (ATCC) and were retained in high-glucose Dulbecco’s Modified Eagle’s Medium (DMEM) (Gibco, CA, USA), supplemented with 10% fetal bovine serum (FBS) (Hyclone, CA, USA), 1% penicillin/streptomycin (Gibco, CA, USA), and 1% MEM non-essential amino acid (Gibco, CA, USA) at 37 °C under 5% CO_2_ conditions. The pHBV1.3 mer DNA encoding wild-type HBV genome was generously contributed by Dr. Jing-hsiung James Ou (University of Southern California). HepAD38 cells were a generous contribution by Dr. Christoph Seeger (Philadelphia, PA) [27]. HepAD38 cells, having tetracycline-responsible promoter that harbored the whole HBV genome, were sustained in Roswell Park Memorial Institute media (RPMI 1640) (Gibco, CA, USA), supplemented with 20% FBS, 1% penicillin/streptomycin (Gibco, CA, USA), and 1% MEM non-essential amino acid (Gibco, CA, USA). They were grown in the presence of 0.5 mg/ml G418 (Invitrogen, CA, USA), and 1 mg/ml tetracycline at 37 °C under 5% CO_2_ conditions [27]. HepG2 cells were grown in 6 well plates and transiently transfected with the plasmid (300 ng) encoding1.3mer HBV genome using TransIT®-LT1 transfection reagent (Mirus; Madison, WI, USA) according to manufacturer’s protocol. Both HepG2 and HepAD38 cells were grown prior to and after treatment with NAC (Millipore Sigma, MO, USA), EGTA-AM (Calbiochem, CA, USA), BAPTA-AM (Abcam: Cambridge, MA, USA), and, Ru360 (EMD Millipore corp; Billerica, MA, USA).

### Antibodies

For western blot, anti-FAM26F C-terminal (ab194946; Abcam, Cambridge, MA, USA) rabbit polyclonal antibody (1:1000 dilution), IRF3 (D83B9; Cell signaling, Danvers, MA, USA) rabbit monoclonal antibody (1:1000 dilution), HBcAg (Santa Cruz Biotechnology, Dallas, TX, USA), GAPDH (FL-335; Santa Cruz Biotechnology, Dallas, TX, USA) rabbit polyclonal antibody (1:1000 dilution), and mouse-anti rabbit IgG HRP-conjugated (Sc-2357; Santa Cruz Biotechnology, Dallas, TX, USA)(1:10000 dilution) were used. The images were quantified by ImageJ software.

### Immunoblotting

For immunoblotting, proteins were extracted from the lysates of both HepAD38 and HepG2/pHBV1.3 cells after 3 and 5 days post HBV induction respectively. Protein Assay kit (Bio-Rad, CA, USA) was used (according to the manufacturer’s instructions) to measure protein concentration. Fifty micrograms of protein was then subjected to SDS-PAGE and transferred thereafter to the nitrocellulose membrane (Thermo Scientific, CA, USA). After successful transfer, the blot was blocked with 5% Bovine serum albumin (BSA) for 1 h. at room temperature followed by three washes with 1XTBS-T buffer (0.05% Tween 20). Further, the blots were incubated with appropriate primary antibodies (1:1000) overnight at 4 °C. The next day, after being washed three times with 1XTBS-T buffer, the blot was incubated with particular horseradish peroxidase (HRP) labeled secondary antibodies (1:10000) at room temperature for 2 h., and then again washed thrice with 1XTBS-T buffer. The membranes were finally incubated with chemiluminescent HRP substrate for 1 min at room temperature. Kodak image station (Digital science, 440) was used to visualize the positive bands by following the manufacturer’s instructions. Quantification of the images was done by ImageJ software.

### Study subjects

Sixty individuals were included in the study who were then divided into 4 groups: controls (*n* = 27), inactive carriers (*n* = 4), recovered cases (*n* = 10), and chronic hepatitis B patients (*n* = 19). Blood samples for the control group were collected from healthy blood donors, whereas those for the inactive carriers, recovered cases, and chronic hepatitis B subjects were taken from the Holy Family Hospital Rawalpindi, Pakistan. The characteristics of each patient like age, sex etc. are given in Table 1.

**Table 1:** Characteristic details of HBV patients in various groups

No.	Patient group	FAM26F expression (fold change)	Viral Load (copies/ml)	Age	Sex	ALT levels
1	Inactive Carriers	0.1339717	128,000	42–52	M	39
2		0.03847326	23,824		M	50
3		0.004487103	23,746		M	46
4		0.000911165	346,899		M	28
5	Recovered cases	0.4061262	7000	22–45	M	29
6		0.4352753	6150		M	28
7		0.2176376	4404		F	25
8		0.000011564	460,000		M	35
9		0.000113896	19,000		F	30
10		0.00158643	38		M	33
11		0.002093308	126,610		M	42
12		0.000455583	2345		F	40
13		0.000523327	46		F	44
14		0.000011564	10		M	27
15	Chronic HBV patients	0.2332582	238,482	11–60	M	80
16		0.03589682	195,000		M	76
17		0.002243551	6,342,857		M	88
18		0.000227791	891,382		F	70
19		0.00837323	3,100,000		M	72
20		0.0078125	4,600,000		M	197
21		0.000345267	4,100,000		M	67
22		0.009618316	389,333		F	13
23		0.02209709	25,821,200		M	28
24		0.1015316	19,764,790		M	38
25		0.000016354	106,629		M	30
26		0.015625	19,000		F	53
27		0.000976563	19,764,790		F	132
28		0.004809158	32,895		M	17
29		0.01269144	3387		F	74
30		0.5358867	1.71E+ 07		M	28
31		0.006345722	223,865		F	132
32		0.03589682	19,000		M	102
33		0.001700294	1,189,725		M	57

The selected individuals fulfilled the inclusion criterion of their respective groups. The criteria were as follows: for HBV inactive carriers: 1) a history of HBsAg being positive for > 4 years, 2) anti-HBe antibody positive, HBeAg negative, 3) no clinically proven symptoms of liver disease, and 4) less than 10^5^ copies/ml of serum HBV DNA; for Chronic Hepatitis B subjects: 1) HBsAg positive for > 6 months or more, 2) Serum HBV DNA > 20,000 IU/mL (in HBeAg-positive patients), and 3) serum HBV DNA between 2000 and 20,000 IU/mL (in HBeAg-negative patients); for Recovered cases: 1) HBsAg negative, 2) hepatitis B core antibody (antiHBc) positive, and 3) anti-HBs positive. Patients who did not meet the above-mentioned criteria were excluded from the study. For control subjects, the criterion was: 1) HBsAg, anti-HBs, HBcAg, anti-HBc or anti-HBc negative, 2) undetectable serum HBV DNA levels, and 3) normal ALT levels.

### RNA extraction

For in vitro experiments, Qiagen RNeasy® mini kit was used for RNA extraction from cell lines as per the manufacturer’s instructions. For in vivo experiments, RNA was extracted from Peripheral Blood Mononuclear Cells (PBMCs) using the Trizol method [28].

### cDNA synthesis

For in vitro study, complementary DNAs were synthesized using Superscript™ III First-Strand Synthesis SuperMix (Invitrogen, CA, USA). For in vivo study, Moloney Murine Leukemia Virus Reverse Transcriptase (M-MLV RT) (Invitrogen, Cat No: 28025013) was used for cDNA synthesis. Both experiments were performed following the respective manufacturer’s instructions. Finally, the cDNA was diluted in the ratio 1:10 for further downstream experiments.

### Real-time qRT-PCR

The RNA level of each gene was quantified through real-time qRT-PCR using DyNAmo HS SYBR Green qPCR kit (#F-410 L; Thermo Scientific, CA, USA). Primer pairs for the target genes were designed using primer3 software [29], which were then optimized by gradient PCR to determine their optimal annealing temperature. The primer pairs used for qRT-PCR were as follows: FAM26F forward primer: 60-TGTTGGGCTGGATCTTGATAG; FAM26F reverse primer: 60-CTGCTGCTTCCTGTTCCAA; IFNB1 forward primer: 60-ATGACCAACAAGTGTCTCCTCC; IFNB1 reverse primer: 60-GCTCATGGAAAGAGCTGTAGTG, GAPDH forward primer: 60-CCTGCACCACCAACTGCTTA; and GAPDH reverse primer: 60-CATGAGTCCTTCCACGATACCA. ABI PRISM 7000 Sequence Detection System (Applied Biosystems) was used to conduct the Real-time qPCR.

### Statistical analysis

All the data are representative of three independent sets of experiments. For statistical analysis of the data, the Student’s t-test and one-way analysis of variance (ANOVA) were performed using Graph-Pad Prism 5.01 software.

## Results

In the in vitro experiments, the expression of FAM26F was determined by both its mRNA as well as protein levels, whereas IRF3 and IFN-β were analyzed only for their protein and mRNA expression respectively. For the in vivo experiments, only FAM26F protein expression was assessed during the study.

### In vitro experiments

#### Positive HBV infection in HepAD38 and HepG2 cell lines

**Fig. 1:**
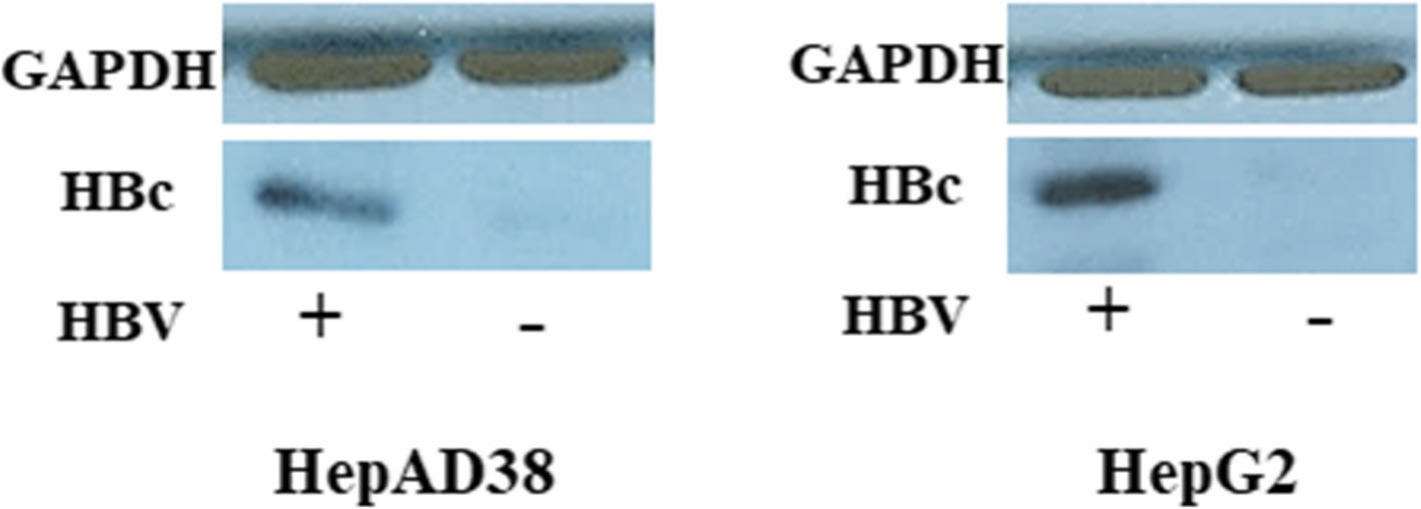
Immunoblot analysis of HBV core protein (HBc) from extracts of HepAD38 and HepG2 cells expressing whole HBV genome and the HBV 1.3mer plasmid respectively. Both cell lines showed successful HBV infection. GAPDH was used as internal control

The first step prior to successive experiments was to confirm the presence of HBV infection in both HepAD38 and HepG2 cell lines. This was achieved by determining the level of HBV core protein (HBc) in infected cells as compared to the uninfected cells (Fig. 1). The results clearly show positive HBV infection in both the cell lines (detailed image in Additional file 1).

#### Reduction in IRF3, FAM26F, and IFN-β expressions post HBV infection

**Fig. 2:**
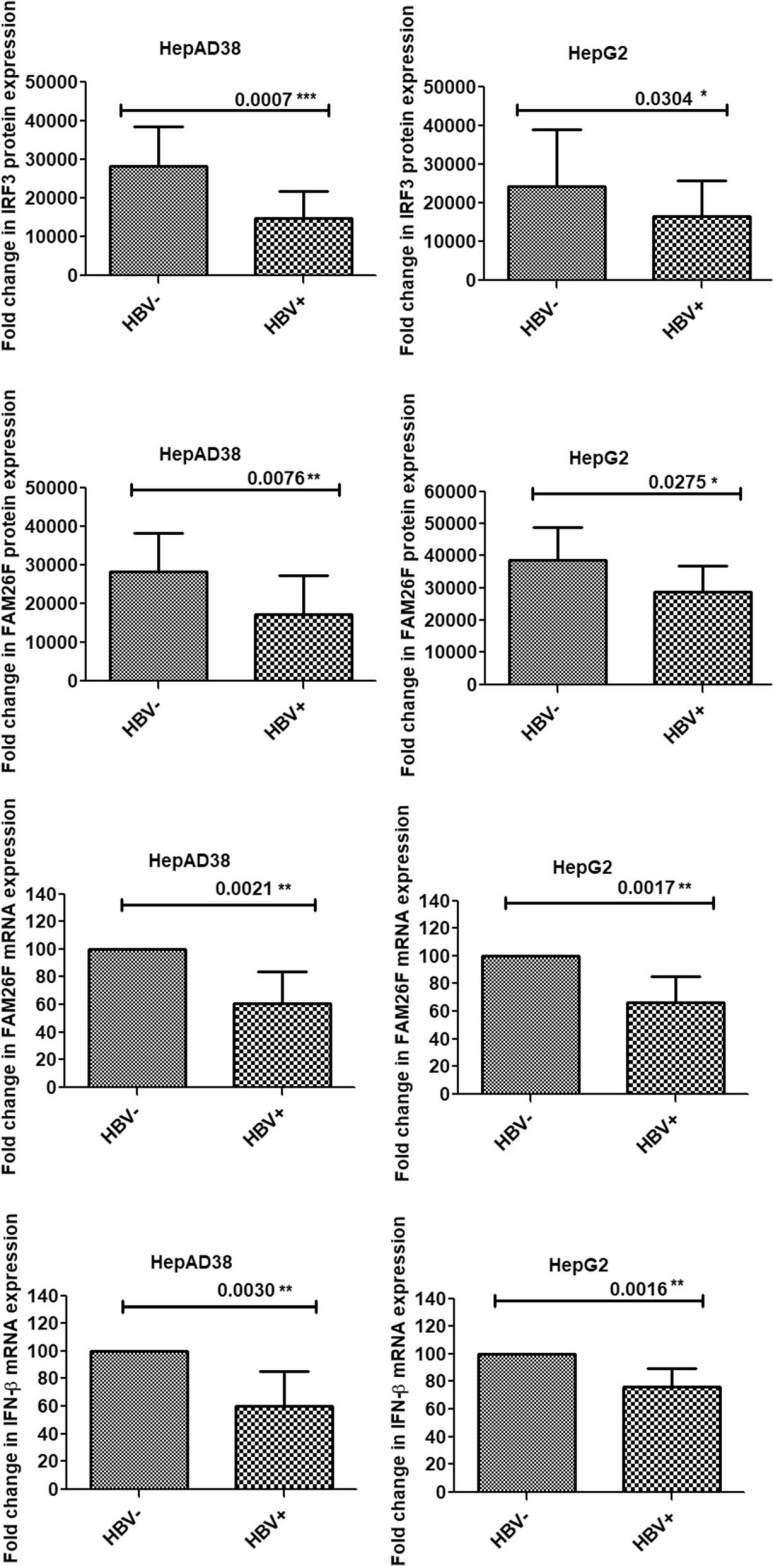
Down-regulation of IRF3, FAM26F, and IFN-β expression post HBV infection. The mRNA and proteins were extracted from HepAD38 and HepG2/pHBV1.3 (300 ng) cells 3 and 5 days post HBV induction respectively. GADPH was used as an internal control. IRF3, FAM26F, and IFN-β expressions were significantly down-regulated as compared to uninfected control cells. All the experiments were performed in triplicates (±SD) and the significance was calculated by Student’s t-test (*P < 0.05, **P < 0.01, ***P < 0.001). HBV- represents HBV Negative group and HBV+ represents HBV Positive group

The results revealed that in both HepAD38 and HepG2 cell lines, the respective expressions of IRF3 (*p* = 0.0007, 0.03), IFN-β (0.003, 0.001), and FAM26F (0.007, 0.02; 0.002, 0.001) were significantly down-regulated as compared to uninfected control cells as depicted in Fig. 2.

#### Effect of antioxidant (NAC) treatment on the IRF3, FAM26F, and IFN-β expressions

**Fig. 3:**
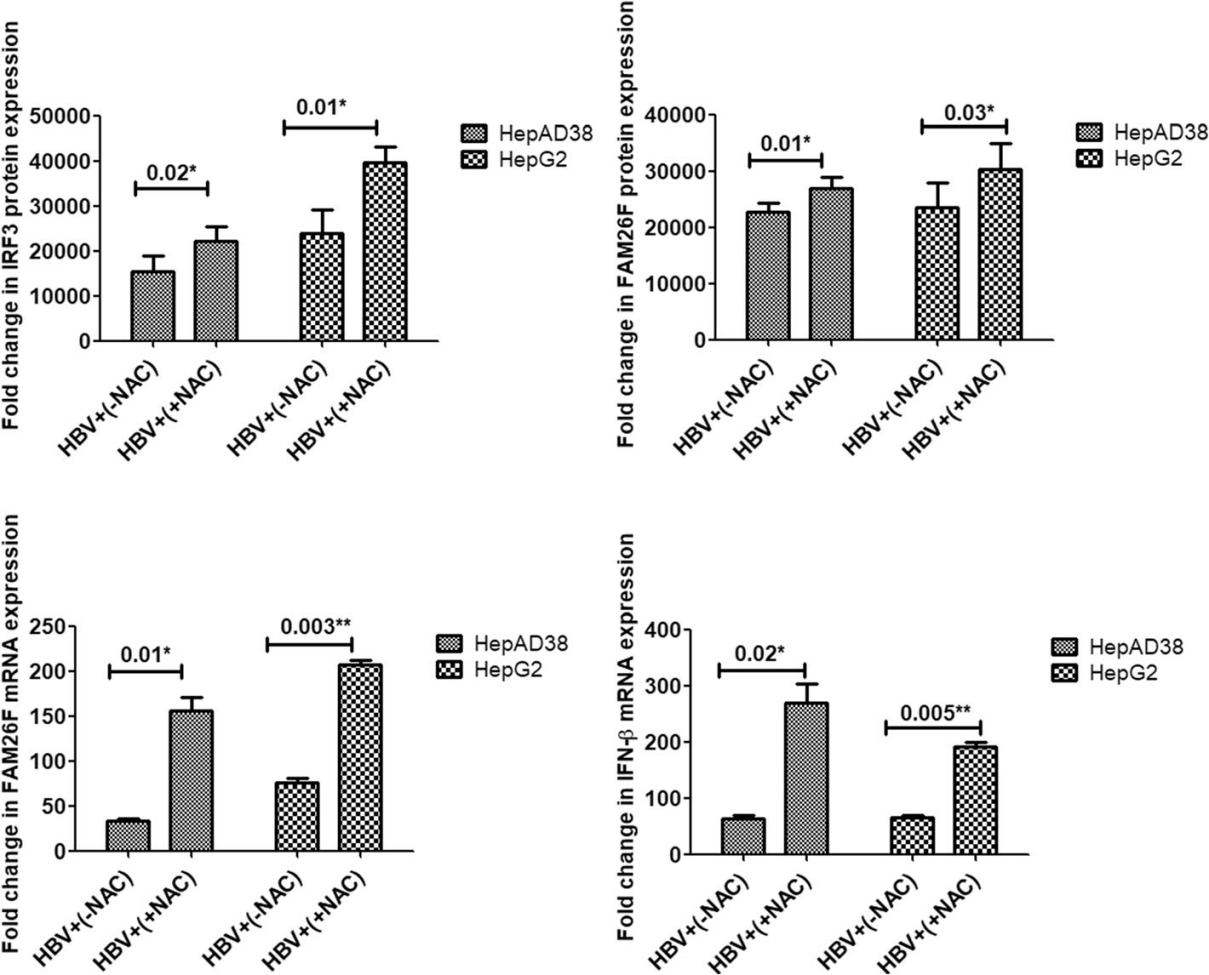
Effect of NAC treatment on expressions of IRF3, FAM26F, and IFN-β. The mRNA and proteins were extracted from HepAD38 and HepG2/pHBV1.3 (300 ng) cells 3 and 5 days post HBV induction respectively. GADPH was used as an internal control. Upregulation of IRF3, FAM26F, and IFN-β expression was seen in HBV+ (+NAC) cells treated with 250 μM NAC for 24 h. Significant differences were seen in both HepAD38 and HepG2/pHBV1.3 cell lines. All the experiments were performed in triplicates (±SD) and the significance was calculated by Student’s t-test(*P < 0.05, **P < 0.01). The X-axis indicates the group without NAC treatment as HBV+(−NAC) and the NAC treated group as HBV+(+NAC), while the Y-axis shows the IRF3, FAM26F, and IFN-β expressions in both cell lines

The effect of NAC on the expressions of IRF3, FAM26F, and IFN-β in HepAD38 and HepG2/pHBV1.3 cell lines were checked prior to and after the treatment of cells with NAC. It was observed that treatment with NAC significantly up-regulated the IRF3 (*p* = 0.02, 0.01), FAM26F (*p* = 0.01, 0.03; *p* = 0.01, 0.003), and IFN-β (*p* = 0.02, 0.005) expressions in both HepAD38 and HepG2/pHBV1.3 cell lines, as shown in Fig. 3.

#### Effect of calcium inhibitors on the IRF3, FAM26F, and IFN-β expressions

Altered Ca^2+^ signaling and elevated Ca^2+^ have been observed in HBV replicating cells [2, 3]. A reversal in this balance is likely to restore Ca^2+^ homeostasis, leading to upregulation of FAM26F production, and ultimately to cell survival. Hence, the next experiments were carried out to check whether or not all the three Ca^2+^ inhibitors used in the study can restore the expression of FAM26F, IRF3, and IFNβ in HBV expressing cells, which was formerly downregulated due to impaired Ca^2+^ regulation.

Treatment with EGTA-AM significantly increased the expression of FAM26F (*p* = 0.02, 0.01; *p* = 0.01, 0.01) and IFN-β (*p* = 0.01, 0.03) in both the cell lines respectively, however, the increase in expression of IRF3 was not found significant (*p* = 0.12, 0.17). Treatment with BAPTA-AM significantly increased the FAM26F protein (*p* = 0.02, 0.005) as well as FAM26F mRNA (*p* = 0.003, 0.01) expression in both the cell lines respectively. A trend in the up-regulation of IRF3 (*p* = 0.12, 0.19) and IFN-β (*p* = 0.08, 0.16) expression was also observed in both HepAD38 and HepG2 cells after BAPTA-AM treatment as compared to non-treated cells, however, this increase was not statistically significant. Similar non-significant trend was observed in the protein expression of IRF3 (*p* = 0.11, 0.23) and FAM26F (*p* = 0.23, 0.21) after Ru360 treatment as depicted in Fig. 5. However, treatment with Ru360 significantly increased the expression of FAM26F (*p* = 0.04, 0.001) and IFN-β mRNA (*p* = 0.003, 0.01) in both the cell lines respectively.

**Fig. 4:**
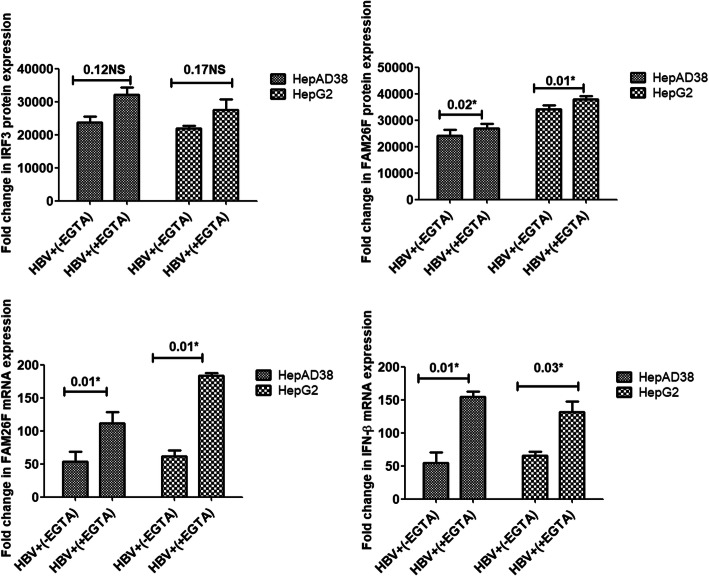
Effect of EGTA-AM treatment on expressions of IRF3, FAM26F, and IFN-β. The mRNA and proteins were extracted from HepAD38 and HepG2/pHBV1.3 (300 ng) cells 3 and 5 days post HBV induction respectively. GADPH was used as an internal control. Upregulation of IRF3, FAM26F, and IFN-β expression was seen in HBV+ (+EGTA) cells treated with 5 μM EGTA for 24 h. Significant differences were seen in both HepAD38 and HepG2/pHBV1.3 cell lines for FAM26F (*p* = 0.02, 0.01; 0.01, 0.01) and IFN-β (*p* = 0.01, 0.03) expression respectively. However, the increase in expression of IRF3 (*p* = 0.12, 0.17) was not found significant. All the experiments were performed in triplicates (±SD) and the significance was calculated by Student’s t-test (*P < 0.05). The X-axis indicates the group without EGTA treatment as HBV+(−EGTA) and the EGTA treated group as HBV+(+EGTA), while the Y-axis shows IRF3, FAM26F, and IFN-β expressions in both the cell lines. NS: non-significant

**Fig. 5:**
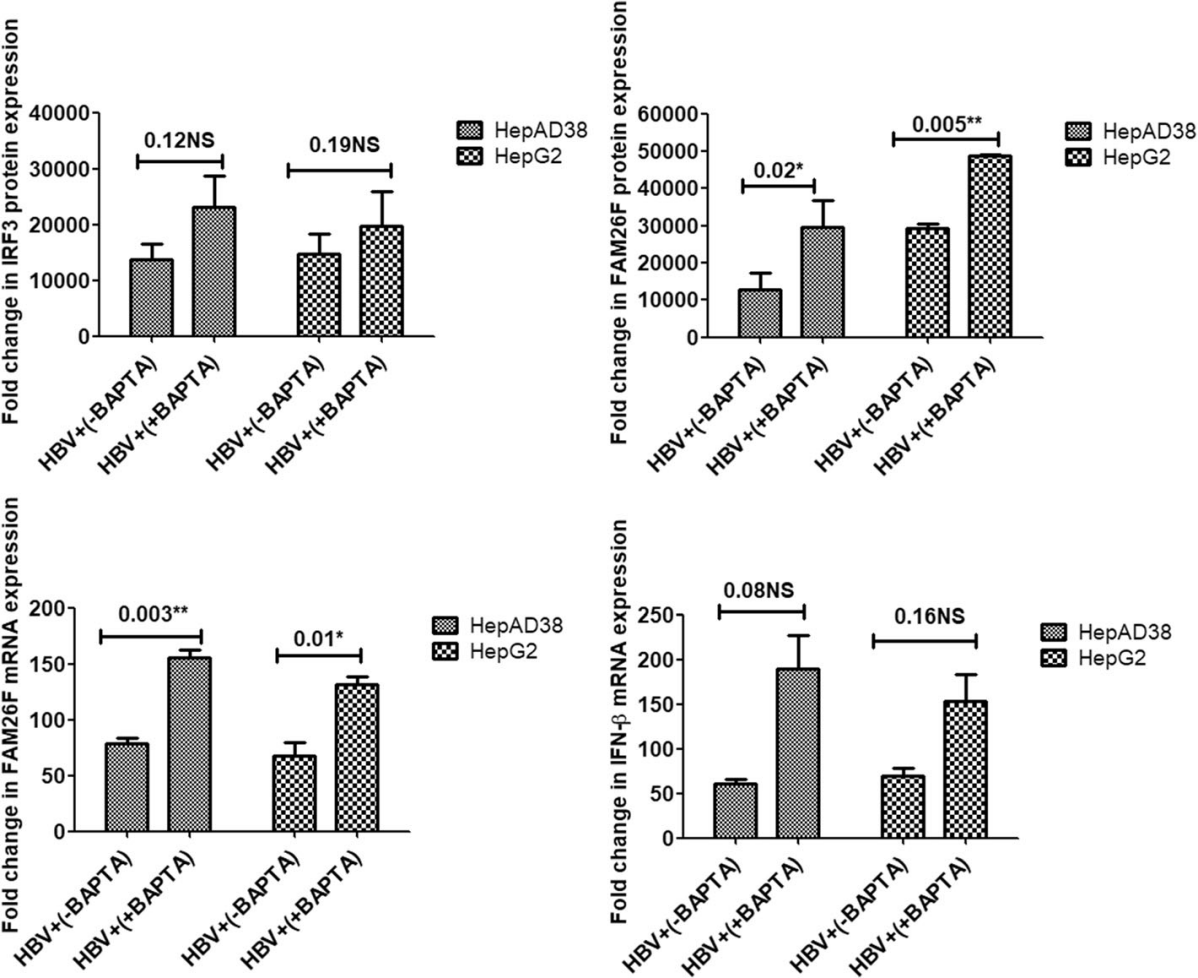
Effect of BAPTA-AM treatment on expressions of IRF3, FAM26F, and IFN-β. The mRNA and proteins were extracted from HepAD38 and HepG2/pHBV1.3 (300 ng) cells 3 and 5 days post HBV induction respectively. GADPH was used as an internal control. Upregulation of IRF3, FAM26F, and IFN-β expression was seen in HBV+ (+BAPTA) cells treated with 5 μM BAPTA for 24 h. Significant differences in the FAM26F expression with *p* = 0.003, 0.01 and *p* = 0.02, 0.005 were seen in both HepAD38 and HepG2/pHBV1.3 cell lines, however, the increase in IRF3 and IFN-β expression was not found significant. All the experiments were performed in triplicates (±SD) and the significance was calculated by Student’s t-test (*P < 0.05, **P < 0.01). The X-axis indicates the group without BAPTA treatment as HBV+(−BAPTA) and the BAPTA treated group as HBV+(+ BAPTA), while the Y-axis shows IRF3, FAM26F, and IFN-β expressions in both cell lines. NS: non-significant

**Fig. 6:**
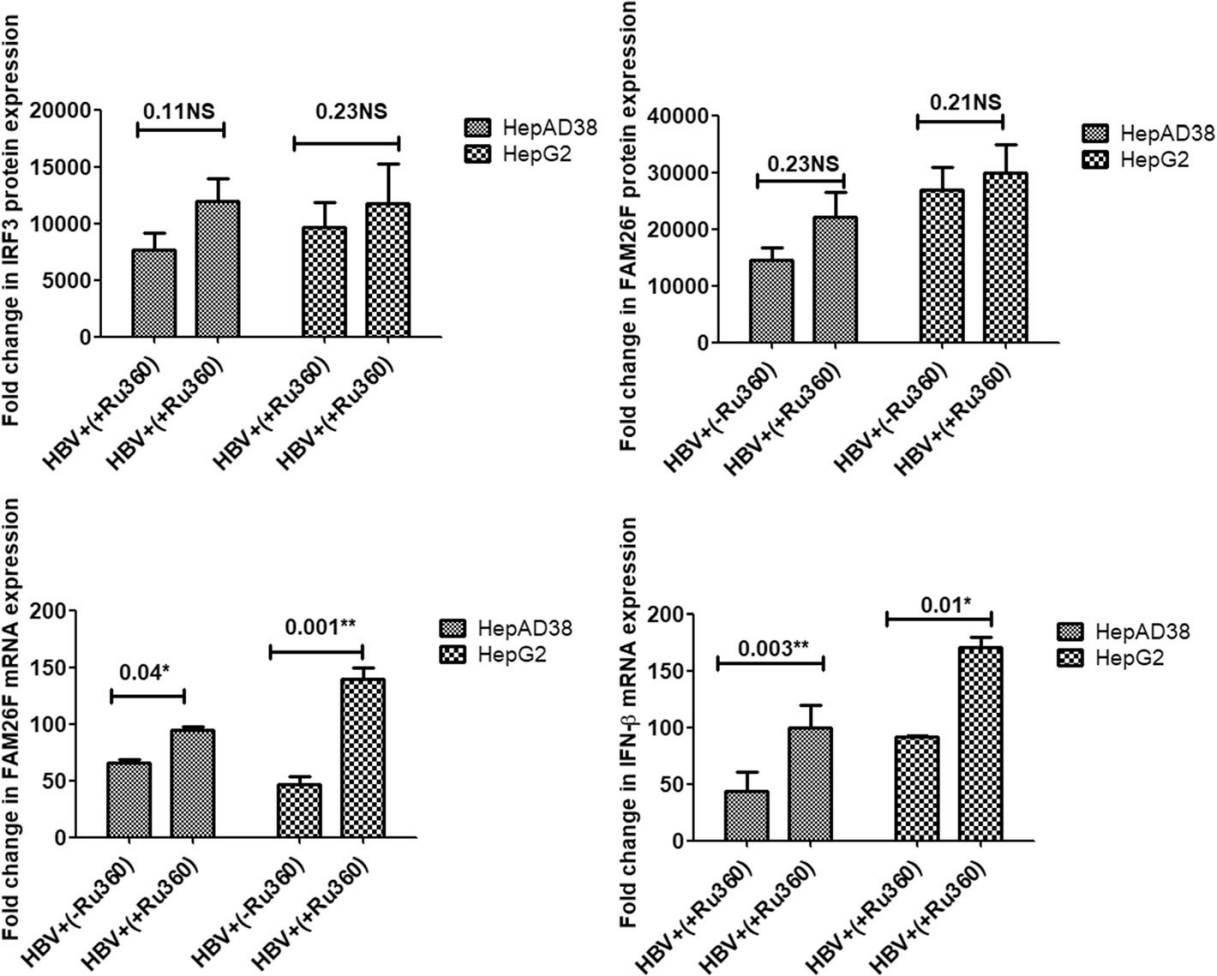
Effect of Ru360 treatment on expressions of IRF3, FAM26F, and IFN-β. The mRNA and proteins were extracted from HepAD38 and HepG2/pHBV1.3 (300 ng) cells 3 and 5 days post HBV induction respectively. GADPH was used as an internal control. Upregulation of IRF3, FAM26F, and IFN-β expression was seen in HBV+ (+Ru360) cells treated with 10 μM Ru360 for 12 h. Significant differences in FAM26F with *p* = 0.04, 0.001 and IFN-β with *p* = 0.003, 0.01 expressions were seen in both HepAD38 and HepG2/pHBV1.3 cell lines respectively, however, an increase in the protein expression of IRF3 and FAM26F was not found significant. All the experiments were performed in triplicates (±SD) and the significance was calculated by Student’s t-test (*P < 0.05, **P < 0.01). The X-axis indicates the group without Ru360 treatment as HBV+(−Ru360) and the Ru360 treated group as HBV+(+ Ru360), while the Y-axis shows the IRF3, FAM26F, and IFN-β expressions in both cell lines. NS: non-significant

Figures 4, 5 and 6 clearly demonstrate a trend in the up-regulation of IRF3, FAM26F, and IFN-β expressions in treated HBV expressing cells as compared to the untreated HBV expressing cells. HBV+(+EGTA) panel in Fig. 3, HBV+(+BAPTA) panel in Fig. 4, and HBV+(+Ru360) panel in Fig. 5 shows an increase in the IRF3, FAM26F, and IFN-β expressions compared to HBV+(−EGTA), HBV+(−BAPTA), and HBV+(−Ru360) respectively.

### In vivo experiments

#### Differential expression and correlation of FAM26F with viral load in various patient groups and uninfected controls

**Fig. 7:**
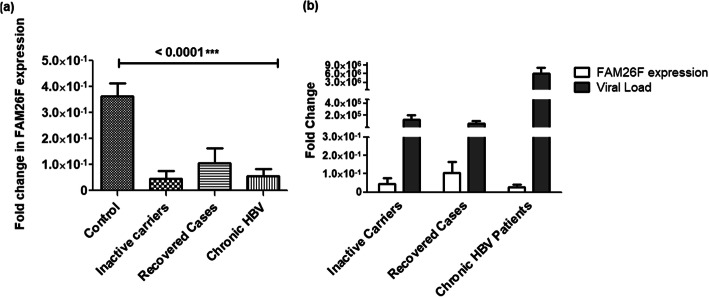
Differential expression of and correlation of FAM26F with viral load among HBV infected groups and uninfected controls. Statistically significant difference of the FAM26F expression was seen between Controls (*n* = 27), Inactive carriers (*n* = 4), Recovered cases (*n* = 10) and Chronic HBV patients (*n* = 19) with *p* = 0.0007. **b** The correlation of FAM26F with viral load of various patient groups demonstrates an inverse relation. Recovered cases with least viral load show maximum FAM26F expression as compared to the Inactive carriers as well as Chronic HBV patient groups. The X-axis indicates the different sample groups, while the Y-axis shows the relative expression values. All the experiments were performed in triplicates (±SD) and the significance was calculated by One-way ANOVA (***P < 0.001)

The expression of FAM26F was also quantified in vivo and compared among various HBV infected patient groups as categorized by their disease progression state. The FAM26F expression was highest in controls and in the patients that had recovered from HBV infection. On the other hand, the FAM26F expression was significantly downregulated in all HBV infected groups as compared to controls. Among different study groups, FAM26F was significantly differentially expressed in samples from HBV patients as well as in controls with *p* = 0.0007 as shown in Fig. 7a. The correlation of FAM26F with viral load of various patient groups demonstrated an inverse relation. Recovered cases with least viral load had maximum FAM26F expression as compared to the Inactive carriers as well as Chronic HBV patient groups (Fig. 7b).

## Discussion

Calcium homeostasis plays a central role in the activation of cells of the immune system by increasing the cytosolic calcium levels through extracellular Ca^2+^ influx and by triggering Ca^2+^ release from the intracellular stores (Endoplasmic Reticulum (ER) and Golgi) [30], ultimately mediating the release of large amounts of ROS [30]. This production of ROS in turn allows the host cell to trigger an efficient RIG-I-mediated IRF-3 activation and downstream antiviral genes [31]. FAM26F, also termed as Calcium homeostasis modulator protein 6 (CALHM6), has recently been recognized as a potent innate immunity modulator, owing to its calcium homeostasis modulator domain [19]. Previously, we have demonstrated that FAM26F is functionally related to calcium-binding proteins, specifically to Thioredoxin, and it was proposed that the FAM26F expression is regulated by cytosolic calcium disturbances [20]. Likewise, the HBV expression has also been shown to be linked with disturbance in calcium homeostasis and up-regulation of ROS levels [2, 3]. Owing to the Ca^2+^ modulation by FAM26F and its interaction with Thioredoxin, it was hypothesized that FAM26F might play a role in Ca^2+^ and ROS regulation post HBV infection. However, no previous study or data is available in this regard.

Hence thecurrent study was designed to decipher the expression of FAM26F in vitro and in vivo upon HBV infection or in HBV infected patients respectively, and the effect of using ROS and calcium inhibitors on the regulation of FAM26F expression. Owing to the significance of IRF3 and IFN-β in mediating antiviral response and their reported involvement in regulating and enhancing FAM26F expression [25, 26], IRF3, and IFN-β were also investigated simultaneously with FAM26F for their expression regulation.

The results of the current study demonstrated that the expression of FAM26F, IRF3, and IFN-β was significantly down-regulated in both HBV replicating (HepAD38) and HBV induced (HepG2) cells as compared to uninfected control cells (Fig. 2). The decreased expression of IRF3, and IFN-β can be attributed to the various strategies that HBV has evolved to evade the host immune system by interrupting IFN-inducing cascades, hence resulting in their decreased expression [32–40]. Interestingly, IRF3 and IFN-β are both found to be essential for inducing the expression of FAM26F. A study involving the activation of NK cells by FAM26F showed that FAM26F induction was dependent on TICAM-1 and IRF-3 activation, as TICAM-1−/−or IRF3−/− knockout mDC was unsuccessful in inducing full NK cytotoxicity [25]. Another study investigating the diverse macrophage activation in response to cytokines identified FAM26F as a responsive gene induced by more than one cytokine (IFN-γ, IFN-β, and IL-10) [26]. Since IRF3 and IFN-β are the upstream players of FAM26F, and their expression is impaired during viral infection, hence FAM26F expression was also found to decrease upon HBV infection. This decrease in FAM26F expression analogous to the decrease in expressions of IRF3 and IFN-β suggests that FAM26F can also serve as a potential marker of HBV infection.

HBV can induce excessive oxidative stress and ROS production [41, 42], causing oxidative damage to hepatocytes and finally leading to the development of liver disease [43]. In the current study, to counter the detrimental effects of ROS, both HepAD38 and HepG2 cell lines were treated with the antioxidant NAC which resulted in an increased expression of FAM26F, IRF3, and IFN-β (Fig. 3). This indicates that NAC treatment somehow downregulated HBV replication, and thus elevated the virus-induced inhibitory effect from these antiviral immune proteins. This is consistent with previous studies which demonstrated similar effects when ROS was inhibited in HBV infected cells.

We further evaluated the effect of calcium chelators on the expression of our target proteins in both HepAD38 and HepG2 cell lines. Treatment with EGTA-AM and BAPTA-AM increased the expression of FAM26F, IFN-β, and IRF3 in both the cell lines (Figs. 4 and 5). HBx acts on stored cytosolic calcium as a fundamental activity for HBV replication [2], thus it was postulated that calcium chelation would result in the decrease of HBV replication, and subsequently diminish its hold on the antiviral immune pathways, resulting in enhanced expression of FAM26F, IRF3, and IFN-β. The results of our study are in accordance with this notion. The significance of using calcium chelators for inhibiting HBV replication has been reported for long, as the compounds that stimulate cytoplasmic calcium accumulation, or mobilization can even replace the requirement for HBx in specifically promoting HBV DNA replication through a variety of pathways [44]. For instance, chelation of cytosolic calcium with BAPTA-AM blocked HBx activation of Pyk2, a critical stimulant of HBV DNA replication [2]. Derivatives of cyclosporine, i.e., cyclosporine A and cyclosporine H which block cytosolic calcium signaling also impaired HBV replication [44]. The blocking of store-operated calcium entry (SOCE) results in the reduction of HBx mediated HBV replication [45]. Another study also demonstrated that the replication of wild-type HBV is inhibited by treating cells with the intracellular calcium chelator BAPTA-AM [2].

Ru360 is a potent and specific inhibitor of mitochondrial calcium uniporter (mCU) [46], the main transporter involved in the uptake of Ca^2+^ into mitochondria [47]. As discussed earlier, HBV replication requires increased cytosolic calcium plateau which is achieved by altering the mitochondrial Ca^2+^ uptake. This mitochondrial uptake of Ca^2+^ dampens Ca^2+^-mediated inhibition of further Ca^2+^ release from the ER and/or Ca^2+^ entry through the SOC channel, thereby prolonging Ca^2+^ entry into the cytosol to elevate cytosolic calcium levels [48–52]. To counter this mechanism and analyze the subsequent effects, both HepAD38 and HepG2 cell lines were treated with Ru360. The results showed a significant upregulation of the FAM26F mRNA as well as protein expression. The expression of IRF3 and IFN-β was also upregulated but not that significantly (Fig. 6). The significant increase in the FAM26F expression indicates the high potential of Ru360, and hence inhibition of mitochondrial Ca^2+^ uptake to counter the HBV infection. Moreover, it also implies that the expression of FAM26F is regulated by more than one pathway. Previous studies have also reported HBV inhibition by blocking the mitochondrial calcium uptake system. For instance, inhibition of the mitochondrial permeability transition pore (MPTP) blocks the HBx-induced increase of cytosolic calcium levels [44, 53]. Another study demonstrated that inhibition of SOCE or mitochondrial calcium uptake blocks the HBx-induced increase in the plateau level of calcium spikes [45]. Furthermore, inhibition of mitochondria1channels with CGP37157 or CsA also blocked HBx activation of HBV DNA replication [2].

In the in vivo experiments, FAM26F was significantly downregulated in all HBV infected groups as compared to controls (Fig. 7a). However, the expression of FAM26F was highest in HBV recovered cases, which was consistent with in vitro results and signified the importance of FAM26F as a critical immune modulator and antiviral agent. Different HBV patient groups demonstrated a significant differential expression of FAM26F as compared to controls. When correlated with viral load in the respective groups, a trend of inverse relation between FAM26F and viral load was observed (Fig. 7b). This might be due to the inhibition of HBV by FAM26F, as was observed previously in a microarray study of HCV patients where FAM26F was identified as one of the various proteins associated with HCV viral clearance [11]. This further emphasizes the importance of FAM26F, and thus merits it for further thorough investigation at molecular level.

## Conclusions

The current study is the first to show the association of FAM26F with HBV. Cumulatively, the results of our study highlight the significance of FAM26F as an innate immune modulator and it is proposed that FAM26F expression could be an early predictive marker for HBV infection. Regardless of whether FAM26F is implicated promptly during the regulation of viral replication or ultimately through the immune resistance; our study has revealed that it is a significant molecule having visible intrinsic worth for further investigation.

## Supplementary Information

**Additional file 1.** Raw immunoblot image of HBV core protein (HBc) from extracts of HepAD38 and HepG2 cells expressing whole HBV genome and the HBV 1.3mer plasmid respectively. GAPDH was used as internal control.

## Data Availability

The raw data of the study consists of CT values for real time quantifications and western blot images for protein analysis, which is not submitted into any repository. This raw data is available with the corresponding author and can be provided on reasonable request.

## Abbreviations

FAM26F: Family with sequence similarity 26, member F
HBV: Hepatitis B virus
NAC: N-acetyl-L-cysteine
BAPTA-AM: 1,2-Bis (2-aminophenoxy)ethane-*N,N,N′,N′*-tetraacetic acid tetrakisacetoxymethyl ester
EGTA-AM: Ethylene-bis (oxyethylenenitrilo) tetraacetic acid Glycol ether diamine tetraaceticacid-acetoxymethyl ester
Ru360: Oxygen-bridged dinuclear ruthenium amine complex
ROS: Reactive oxygen species
HCC: Hepatocellular carcinoma
HCV: Hepatitis C Virus
Ca_hom-mod: Calcium homeostasis modulator
CALHM6: Calcium homeostasis modulator protein 6
VUE: Villitis of unknown etiology
ATCC: American Type Culture Collection
DMEM: Dulbecco’s Modified Eagle’s Medium
FBS: Fetal bovine serum
RPMI 1640: Roswell Park Memorial Institute media
BSA: Bovine serum albumin
HRP: Horseradish peroxidase
PBMCs: Peripheral Blood Mononuclear Cells
M-MLV RT: Moloney Murine Leukemia Virus Reverse Transcriptase
ANOVA: One-way analysis of variance
ER: Endoplasmic Reticulum
CTHRC1: Collagen triple helix repeat containing 1
MMP9: Matrix metalloproteinase 9
SOCE: Store-operated calcium entry
SOC: Store-operated calcium
mCU: Mitochondrial calcium uniporter
MPTP: Mitochondrial permeability transition pores
VDAC: Voltage-dependent anion channel
