# Supplementary material for: Effect of oxidative stress and calcium deregulation on FAM26F (CALHM6) expression during hepatitis B virus infection
Source: BMC Infect Dis. 2021 Feb 27;21:228. doi: 10.1186/s12879-021-05888-0 (PMC7913464; doi:10.1186/s12879-021-05888-0)
Supplement: Supplementary file 1 — Additional file 1. Raw immunoblot image of HBV core protein (HBc) from extracts of HepAD38 and HepG2 cells expressing whole HBV genome and the HBV 1.3mer plasmid respectively. GAPDH was used as internal control. [file 12879_2021_5888_MOESM1_ESM.pdf]

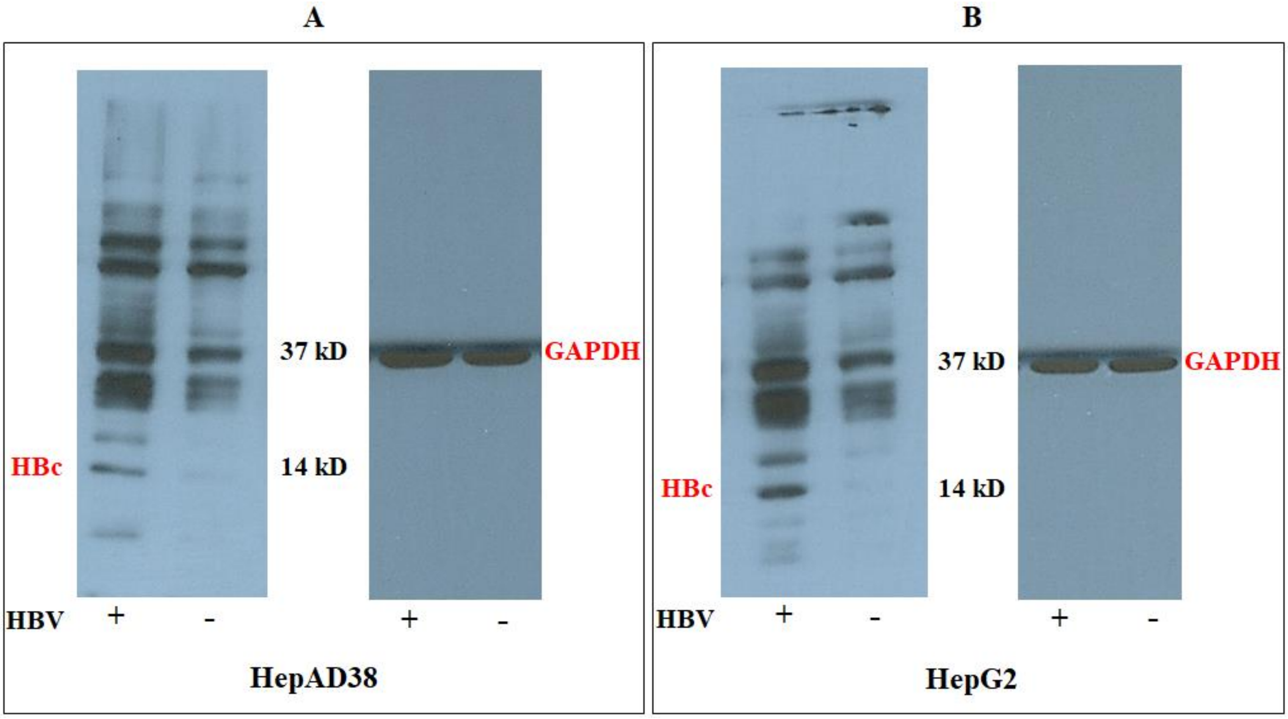

Additional file 1: Raw immunoblot image of HBV core protein (HBc) from extracts of (A) HepAD38 and (B) HepG2 cells expressing whole HBV genome and the HBV 1.3mer plasmid respectively. GAPDH was used as internal control. More than one band for HBcAg in each lane is indicative of the presence of Hep B HBcAg dimers, trimers and tetramers.
